# *In Vitro* Evaluation of the Activity of Aztreonam-Avibactam against 341 Recent Clinical Isolates of Anaerobes

**DOI:** 10.1128/Spectrum.01908-21

**Published:** 2021-12-15

**Authors:** David R. Snydman, Laura McDermott

**Affiliations:** a Department of Medicine, Tufts Medical Center, Boston, Massachusetts, USA; b Stuart B. Levy, MD Center for the Integrated Management of Antimicrobial Resistance, Tufts University School of Medicine, Boston, Massachusetts, USA; University of Texas Southwestern Medical Center

**Keywords:** anaerobes, aztreonam-avibactam, *in vitro* activity

## Abstract

Aztreonam-avibactam is under clinical development for multidrug-resistant Gram-negative infections. We evaluated *in vitro* activity against 341 recent clinical isolates. The addition of avibactam to aztreonam had no effect on the anaerobic activity of aztreonam.

**IMPORTANCE** This work shows that aztreonam-avibactam lacks activity against anaerobic organisms.

## OBSERVATION

The combination of aztreonam-avibactam is being developed for treatment of serious Gram-negative infections due to multidrug-resistant Gram-negative organisms (NCT 01689201) and is being used in combination with metronidazole for intraabdominal infections (1). We tested the *in vitro* activity of aztreonam-avibactam against a broad range of recent clinical anaerobic isolates.

A total of 341 anaerobic clinical isolates from 2016 to 2020 were included in the evaluation. The isolates were referred from specimens collected from patients at Tufts Medical Center from 2016 to 2020. Some isolates were part of a surveillance study for susceptibility of specific organisms referred from other medical centers (2). The identification of all isolates was confirmed previous to susceptibility testing using API20A (bioMérieux, Durham, NC) and/or standard methodology when applicable (3).

The *in vitro* activity of aztreonam-avibactam was compared to that of the following agents: ampicillin-sulbactam, meropenem, metronidazole, moxifloxacin, piperacillintazobactam, clindamycin, and tigecycline. Aztreonam alone and avibactam alone were used as controls. The antimicrobials were solubilized following the manufacturer’s instructions. Stock solutions, 10 to 20 times the desired test concentration, were prepared and kept frozen at −80°C until the day of testing. MICs were determined by agar dilution following CLSI recommendations (4). Table 1 lists the antimicrobials, the range of concentrations tested, and the recommended breakpoints for resistance by the indicated agency. For Clostridioides difficile, the resistance rates based on EUCAST breakpoints are listed when CLSI/FDA breakpoints are not available (5, 6). For tigecycline, a breakpoint of 16 μg/mL was used. This is the breakpoint recommended by the FDA (https://www.accessdata.fda.gov/drugsatfda_docs/label/2013/021821s026s031lbl.pdf).

**TABLE 1:** Antimicrobial agent and breakpoints for resistance used in the study

Antibiotic	Testing range (μg/mL)	Breakpoint for Resistance (μg/mL)
Aztreonam-avibactam^*a*^	0.12–64	NA^*b*^
Aztreonam	0.12–64	NA
Avibactam	0.12–64	NA
Ampicillin:sulbactam 2:1	0.5–128	≥32/16
Piperacillin-tazobactam^*a*^	0.5–256	≥128/4
Clindamycin	0.5–128	≥8
Meropenem	0.12–64	≥16
Metronidazole	1–16	≥32
Moxifloxacin	0.06–64	≥8
Tigecycline^*c*^	0.06–64	≥16
Vancomycin (C. difficile only)^*d*^	0.5–64	>2

a Avibactam and tazobactam were tested at a constant concentration of 4 μg/mL.

b No anaerobic interpretive breakpoint has been established for this agent.

c FDA interpretive breakpoint listed.

d EUCAST interpretive breakpoint listed.

Standardized testing of the isolates was performed at the Special Studies Laboratory at Tufts Medical Center. After arrival of the referred isolate, purity and identification were confirmed using rapid methodology. The isolates were kept using brain heart infusion supplemented (BHIS) agar slants until tested and later stored frozen in skim milk at −80°C for future reference.

The MICs of the isolates were determined using CLSI-recommended agar dilution methodology against the panel of antibiotics stated above. The medium used was brucella agar supplemented with 5 mg hemin, 1 mg vitamin K_1_ per liter, and 5% (vol/vol) lysed sheep blood. The plates were prepared freshly on the morning of the test. Serial 2-fold dilutions of the antibiotics were added to the molten agar, poured into round petri dishes, and allowed to solidify and dry. Non-antibiotic-containing controls were prepared in the same manner. The test organisms were suspended in brain heart infusion supplemented broth (BHIS) to achieve the density of a 0.5 McFarland standard (∼10^8^ CFU/mL) and distributed accordingly into a Steers replicating block that deposited the inocula onto the test plates. The inocula density on the agar surface was ∼10^5^ CFU/spot (∼10^4^ CFU/spot for C. difficile). The plates were incubated for 48 h at 35° to 37°C in an anaerobic chamber with an atmosphere of 85% N_2_, 5% CO_2_, and 10%H_2_. After incubation, the plates were examined against a dark, nonreflecting background and the MICs endpoint was read at the concentration where a marked reduction occurred in the appearance of growth on the test plate compared to that of growth on the control plate. For all testing runs, the following anaerobic quality control organisms were used: Bacteroides fragilis ATCC 25285, Bacteroides thetaiotaomicron ATCC 29741, and Clostridioides difficile ATCC 700057. Klebsiella pneumonia ATCC 700603 and Pseudomonas aeruginosa ATCC 27853 were also included in each run as the control for aztreonam and aztreonam-avibactam. Repeat testing was required if the MIC of a control organism fell outside the CLSI-specified ranges. Quality control values were all within the acceptable range for all testing performed for this study. For all *in vitro* testing, the MICs of the control organisms, B. fragilis ATCC 25285 and B. thetaiotaomicron ATCC 29741, were within the range specified by CLSI and FDA for each agent.

A summary of the comparative activities of the antimicrobial agents against all the species within the group is shown in Table 2. The addition of avibactam to aztreonam had no effect on the lack of activity of aztreonam against a broad variety of anaerobic organisms (4–6) (https://www.accessdata.fda.gov/drugsatfda_docs/label/2013/021821s026s031lbl.pdf).

**TABLE 2:** *In vitro* activity of aztreonam-avibactam against 341 anaerobic organisms from 2016 to 2020 and 9 comparators

Species	Antimicrobial agent	MIC range (μg/mL)	MIC_50_		MIC_90_	Percent resistant^*a*^
B. fragilis group (*n *= 201)	Aztreonam-avibactam	32 to >64	>64		>64	NA^*a*^^*b*^
	Aztreonam	32 to >64	>64		>64	NA
	Avibactam	32 to >64	>64		>64	NA
	Piperacillin-tazobactam	≤0.5–256	4		16	0.5%
	Ampicillin:sulbactam	≤0.5 to >128	4		16	3.0%
	Tigecycline	≤0.06–32	1		4	2.0%
	Moxifloxacin	0.25–64	4		32	46.5%
	Meropenem	≤0.12–128	0.25		2	2.5%
	Clindamycin	≥0.5 to >128	4		>128	46.5%
	Metronidazole	≤1–2	≤1		2	0.0%
B. fragilis (*n *= 59)	Aztreonam-avibactam	32 to >64	>64		>64	NA
	Aztreonam	32 to >64	>64		>64	NA
	Avibactam	32 to >64	>64		>64	NA
	Piperacillin-tazobactam	≤0.5–256	2		16	1.7%
	Ampicillin:sulbactam	≤0.5 to >128	2		16	1.7%
	Tigecycline	0.25–32	0.5		8	3.4%
	Moxifloxacin	0.5–64	2		16	35.6%
	Meropenem	≤0.12–128	0.25		4	6.8%
	Clindamycin	≤0.5 to >128	2		>128	28.8%
	Metronidazole	≤1–2	≤1		2	0.0%
Bacteroides distasonis (*n *= 22)	Aztreonam-avibactam	64 to >64	>64		>64	NA
	Aztreonam	32 to >64	>64		>64	NA
	Avibactam	All >64	>64		>64	NA
	Piperacillin-tazobactam	≤0.5–64	4		32	0.0%
	Ampicillin:sulbactam	2–32	4		32	15.0%
	Tigecycline	0.12–8	1		4	0.0%
	Moxifloxacin	1–64	2		32	40.0%
	Meropenem	≤0.12–4	0.25		2	0.0%
	Clindamycin	≤0.5 to >128	>128		>128	65.0%
	Metronidazole	≤1–2	≤1		2	0.0%
Bacteroides ovatus (*n *= 19)	Aztreonam-avibactam	All >64	>64	>64		NA
	Aztreonam	All >64	>64	>64		NA
	Avibactam	All >64	>64	>64		NA
	Piperacillin-tazobactam	4–32	8	16		0.0%
	Ampicillin:sulbactam	1–8	16	8		0.0%
	Tigecycline	0.5–8	0.5	8		0.0%
	Moxifloxacin	1–64	2	64		31.3%
	Meropenem	≤0.12–8	2	1		0.0%
	Clindamycin	≤0.5 to >128	2	>128		18.8%
	Metronidazole	≤1–2	≤1	2		0.0%
Bacteroides thetaiotaomicron (*n *= 39)	Aztreonam-avibactam	32 to >64	>64	>64		NA
	Aztreonam	32 to >64	>64	>64		NA
	Avibactam	32 to >64	>64	>64		NA
	Piperacillin-tazobactam	≤0.5–32	8	16		0.0%
	Ampicillin:sulbactam	≤0.5–32	2	16		2.6%
	Tigecycline	≤0.06–16	1	8		5.1%
	Moxifloxacin	0.25–32	8	32		61.5%
	Meropenem	≤0.12–4	0.25	2		0.0%
	Clindamycin	≤0.5 to >128	>128	>128		64.1%
	Metronidazole	≤1–2	2	2		0.0%
Bacteroides uniformis (*n *= 21)	Aztreonam-avibactam	64 to >64	>64	>64		NA
	Aztreonam	All >64	>64	>64		NA
	Avibactam	64 to >64	>64	>64		NA
	Piperacillin-tazobactam	≤0.5–32	2	32		0.0%
	Ampicillin:sulbactam	≤0.5–16	2	16		0.0%
	Tigecycline	0.25–8	1	4		0.0%
	Moxifloxacin	≤0.5–32	4	32		44.4%
	Meropenem	≤0.12–4	0.25	2		0.0%
	Clindamycin	≤0.5 to >128	4	>128		50.0%
	Metronidazole	≤1–2	≤1	2		0.0%
Bacteroides vulgatus (*n *= 23)	Aztreonam-avibactam	32 to >64	>64	>64		NA
	Aztreonam	32 to >64	>64	>64		NA
	Avibactam	All >64	>64	>64		NA
	Piperacillin-tazobactam	≤0.5–32	4	8		0.0%
	Ampicillin:sulbactam	1–32	8	16		4.3%
	Tigecycline	0.25–8	0.5	4		0.0%
	Moxifloxacin	2–64	32	64		91.3%
	Meropenem	≤0.12–4	0.5	2		0.0%
	Clindamycin	≤0.5 to >128	>128	>128		65.2%
	Metronidazole	≤1–2	≤1	2		0.0%
Bacteroides caccae (*n *= 12)	Aztreonam-avibactam	64 to >64	>64	>64		NA
	Aztreonam	64 to >64	>64	>64		NA
	Avibactam	64 to >64	>64	>64		NA
	Piperacillin-tazobactam	≤0.5–4	4	4		0.0%
	Ampicillin:sulbactam	1–8	2	8		0.0%
	Tigecycline	0.5–4	2	4		0.0%
	Moxifloxacin	0.12–32	0.25	8		16.6%
	Meropenem	0.25–16	1	4		8.3%
	Clindamycin	≤0.5 to >128	16	>128		58.3%
	Metronidazole	≤1–2	≤1	2		0.0%
Misc. B. fragilis group (*n *= 6) (*n *= 3 Bacteroides eggerthii, *n *= 1 Bacteroides dorei, *n *= 1 Bacteroides splanchnicus, *n *= 1 Bacteroides intestinalis)	Aztreonam-avibactam	All >64				
	Aztreonam	All >64				
	Avibactam	All >64				
	Piperacillin-tazobactam	≤0.5–32				
	Ampicillin:sulbactam	2–16				
	Tigecycline	0.25–4				
	Moxifloxacin	0.25–32				
	Meropenem	1–8				
	Clindamycin	≤0.5 to >128				
	Metronidazole	≤1–2				
*Prevotella* spp. (*n *= 15)	Aztreonam-avibactam	32 to >64	64	64		NA
	Aztreonam	16 to >64	64	>64		NA
	Avibactam	64 to >64	>64	>64		NA
	Piperacillin-tazobactam	≤0.5–8	≤0.5	1		0.0%
	Ampicillin:sulbactam	≤0.5–8	1	4		0.0%
	Tigecycline	≤ 0.06–2	0.25	2		0.0%
	Moxifloxacin	0.5–64	1	16		20.0%
	Meropenem	≤ 0.12–2	≤0.12	0.25		0.0%
	Clindamycin	≤0.5–128	≤0.5	128		33.0%
	Metronidazole	≤1–2	≤1	2		0.0%
*Fusobacterium* spp. (*n *= 15)	Aztreonam-avibactam	32 to >64	64	>64		NA
	Aztreonam	32 to >64	64	>64		NA
	Avibactam	32 to >64	64	>64		NA
	Piperacillin-tazobactam	≤0.5–4	≤0.5	≤0.5		0.0%
	Ampicillin:sulbactam	≤0.5–2	≤0.5	1		0.0%
	Tigecycline	≤0.06–0.5	0.5	2		0.0%
	Moxifloxacin	0.25–4	1	4		0.0%
	Meropenem	≤0.12–0.5	≤0.12	0.25		0.0%
	Clindamycin	≤0.5–32	≤0.5	4		6.6%
	Metronidazole	≤1–2	≤1	2		0.0%
C. difficile (*n *= 40)	Aztreonam-avibactam	All >64	>64	>64		NA
	Aztreonam	All >64	>64	>64		NA
	Avibactam	All >64	>64	>64		NA
	Piperacillin-tazobactam	≤0.5–16	2	8		0.0%
	Ampicillin:sulbactam	≤0.5–4	≤0.5	2		0.0%
	Tigecycline	≤0.06–0.5	≤0.06	0.25		0.0%
	Moxifloxacin	≤0.5–64	4	32		22.5%
	Meropenem	≤0.12–2	1	2		0.0%
	Clindamycin	≤0.5 to >128	2	>128		25.0%
	Metronidazole	0.25–2	≤1	≤1		0.0%
	Vancomycin^*c*^	≤0.5–4	2	2		5.0%
Clostridium perfringens (*n *= 15)	Aztreonam-avibactam	64 to >64	>64	>64		NA
	Aztreonam	All >64	>64	>64		NA
	Avibactam	All >64	>64	>64		NA
	Piperacillin-tazobactam	≤0.5–1	≤0.5	≤0.5		0.0%
	Ampicillin:sulbactam	≤0.5–2	≤0.5	1		0.0%
	Tigecycline	0.12–2	0.5	2		0.0%
	Moxifloxacin	0.25–4	1	4		0.0%
	Meropenem	≤0.12–4	≤0.12	0.25		0.0%
	Clindamycin	≤0.5–128	1	64		0.1%
	Metronidazole	≤1–2	≤1	2		0.0%
	Vancomycin	≤0.5–2	0.5	1		0.0%
*Clostridium* spp. (*n *= 7 C. innocuum, *n *= 3 C. beijennckii, *n *= 2 C. ramosum), *n *= 1 C. clostridioforme)	Aztreonam-avibactam	64 to >64	>64	>64		NA
	Aztreonam	64 to >64	>64	>64		NA
	Avibactam	All >64	>64	>64		NA
	Piperacillin-tazobactam	≤0.5–16	2	16		0.0%
	Ampicillin:sulbactam	≤0.5–2	≤0.5	2		0.0%
	Tigecycline	≤0.06–1	0.12	0.5		0.0%
	Moxifloxacin	0.5 to >64	2	4		10.0%
	Meropenem	≤0.12–4	1	2		0.0%
	Clindamycin	≤0.5–128	4	128		45.0%
	Metronidazole	All <1	≤1	≤1		0.0%
	Vancomycin	1–16	2	8		0.0%
*Peptostreptococcus* spp. (*n *= 10)	Aztreonam-avibactam	32 to >64	32	>64		NA
	Aztreonam	32 to >64	64	>64		NA
	Avibactam	32 to >64	32	>64		NA
	Piperacillin-tazobactam	≤0.5–1	≤0.5	≤0.5		0.0%
	Ampicillin:sulbactam	≤0.5–2	≤0.5	≤0.5		0.0%
	Tigecycline	≤0.06–0.5	0.25	0.25		0.0%
	Moxifloxacin	0.12–4	0.5	2		20.0%
	Meropenem	≤0.12–0.5	≤0.12	≤0.12		0.0%
	Clindamycin	≤0.5–8	1	2		10.0%
	Metronidazole	≤1–2	≤1	2		0.0%
	Vancomycin	≤0.5–1	1	1		0.0%
*Actinomyces* spp. (*n *= 9)	Aztreonam-avibactam	32 to >64				
	Aztreonam	32 to >64				
	Avibactam	32 to >64				
	Piperacillin-tazobactam	≤0.5–8				
	Ampicillin:sulbactam	≤0.5–2				
	Tigecycline	0.25–1				
	Moxifloxacin	2–16				
	Meropenem	≤0.12–2				
	Clindamycin	≤0.5–32				
	Metronidazole	≤1–4				
	Vancomycin	1–2				
*Bifidobacterium* spp. (*n *= 5)	Aztreonam-avibactam	64 to >64				
	Aztreonam	32 to >64				
	Avibactam	All >64				
	Piperacillin-tazobactam	All ≤0.5				
	Ampicillin:sulbactam	All ≤0.5				
	Tigecycline	0.25–0.5				
	Moxifloxacin	0.5–2				
	Meropenem	All ≤0.12				
	Clindamycin	≤0.5–1				
	Metronidazole	8–16				
	Vancomycin	≤0.5–1				
*Cutibacterium* spp. (*n *= 6)	Aztreonam-avibactam	32 to >64				
	Aztreonam	32 to >64				
	Avibactam	64 to >64				
	Piperacillin-tazobactam	≤0.5–1				
	Ampicillin:sulbactam	≤0.5–1				
	Tigecycline	≤0.06–0.5				
	Moxifloxacin	1–4				
	Meropenem	≤0.12–1				
	Clindamycin	1–16				
	Metronidazole	All >32				
	Vancomycin	1–2				
*Lactobacillus* spp. (*n *= 5)	Aztreonam-avibactam	All >64				
	Aztreonam	All >64				
	Avibactam	All >64				
	Piperacillin-tazobactam	≤0.5–2				
	Ampicillin:sulbactam	1–4				
	Tigecycline	0.25–2				
	Moxifloxacin	1–2				
	Meropenem	2–8				
	Clindamycin	1–32				
	Metronidazole	All >32				
	Vancomycin	2–64				

a Percent resistant was calculated using CLSI-recommended breakpoints, except for tigecycline, where the FDA breakpoint was used.

b NA: CLSI-recommended breakpoints for anaerobic bacteria have not been established.

c The EUCAST-recommended resistance breakpoint for vancomycin of >2 μg/mL was used for C. difficile only.

The lack of activity of the combination is consistent with the fact that avibactam does not inhibit the metalloenzyme (*cfi*A gene) produced by Bacteroides fragilis group. From a clinical standpoint, absence of activity for aztreonam/avibactam means that the addition of specific antianaerobic therapy will be necessary for treatment of mixed aerobic and anaerobic infections.
